# LncRNA DSCAM-AS1 promotes colorectal cancer progression by acting as a molecular sponge of miR-384 to modulate AKT3 expression

**DOI:** 10.18632/aging.103243

**Published:** 2020-05-26

**Authors:** Bo Li, Hai Sun, Jiayu Zhang

**Affiliations:** 1 Department of Gastrointestinal Colorectal and Anal Surgery, China-Japan Union Hospital of Jilin University, Changchun 130021, P.R. China; 2 Department of Anesthesiology, China-Japan Union Hospital of Jilin University, Changchun 130021, P.R. China

**Keywords:** colorectal cancer, lncRNA, DSCAM-AS1, miR-384, AKT3

## Abstract

Down Syndrome Cell Adhesion Molecule antisense1 (DSCAM-AS1), a novel long non-coding RNA (lncRNA), reportedly contributes to the development and progression of several cancers. There is a lack of information on its biological role and regulatory mechanism with respect to colorectal cancer (CRC). Here, we discovered that the expression of DSCAM-AS1 exhibited a significant upregulation in CRC tissues and cell lines in comparison with the corresponding control. Increased DSCAM-AS1 expression was associated with poor prognosis for those diagnosed with CRC. Loss-of function assay illustrated that knockdown of DSCAM-AS1 resulted in significant inhibition of cell proliferation, invasion and migration *in vitro*, and impaired tumor growth *in vivo*. MicroRNA-384(miR-384) was directly targeted by DSCAM-AS1 in CRC cells, and repression of DSCAM-AS1 inhibited the expression of AKT3, a known target of miR-384 in CRC. In addition, repression of miR-384 or overexpression of AKT3 could partially rescue the inhibitory effect of DSCAM-AS1 knockdown on CRC progression. In summary, DSCAM-AS1 exerted an oncogenic role in CRC by functioning as a competing endogenous RNA of miR-384 to bring about regulation of AKT3 expression. These results implied that DSCAM-AS1 might be a novel therapeutic target for patients suffering from CRC.

## INTRODUCTION

Colorectal cancer (CRC), a commonly diagnosed digestive malignant tumor, is the third highest reason for tumor-associated mortality around the world [1]. In spite of great efforts being made to develop effective strategies against CRC, the outcomes for patients is unsatisfactory [2, 3]. Thus, studying the potential mechanisms involved in occurrence and progression of CRC is crucial to explore novel targets for diagnosis and treatment of this disease.

Spanning over 200 nucleotides in length, long non-coding RNA (lncRNA) are a class of RNA transcripts with little protein-coding potential [4]. Emerging evidence demonstrates that lncRNAs play a crucial regulatory role among various cellular processes, like cell proliferation, invasion, apoptosis and cycle [5, 6]. Studies show that lncRNAs are associated with initiation and development of various cancers [7]. Many lncRNAs are verified to play tumor suppressor or oncogenic role in the progression of CRC [8, 9], suggesting that lncRNAs could serve as a diagnosis marker and therapy agent.

Down Syndrome Cell Adhesion Molecule (DSCAM) antisense (DSCAM-AS1), (DSCAM-AS1), a novel lncRNA, has been reported to be upregulated and function as oncogenic lncRNA in hepatocellular carcinoma [10], non-small lung cancer [11], ovarian cancer [12], melanoma [13] and breast cancer [14–16]. Despite recently studies demonstrated that DSCAM-AS1 expression was upregulated in CRC and was involved in CRC proliferation and invasion [17, 18], the function and underlying mechanism of DSCAM-AS1 in CRC progression remains largely unknown.

Studies show that LncRNAs can depict competing endogenous RNAs (ceRNAs) or natural microRNA (miRNA) sponges that bring about modulation of miRNAs [19, 20]. MiRNAs (small non-coding RNAs: 18-25 nucleotides), bring about inhibition of translation or degradation of target mRNAs when it binds to the 3′-untranslated regions (3′-UTR) of target genes [21]. MicroRNAs (miRNAs) was reported to play crucial roles in multiple cancer processes [22]. Nevertheless, it remains largely unclear whether DSCAM-AS1 can serve as ceRNA of miRNAs to regulate CRC progression.

In our study, analysis of DSCAM-AS1 expression in CRC tissues and its relationship with clinicopathologic characteristics of CRC patients was carried out. Functional roles of DSCAM-AS1 on CRC cell growth and metastasis were determined by numerous experiments. Moreover, the regulatory mechanism of DSCAM-AS1 in CRC was investigated by confirming whether it serves as a ceRNA of miRNA to modulate CRC progression.

## RESULTS

### Upregulation of DSCAM-AS1 and its correlation with poor prognosis in patients with CRC

**Figure 1:**
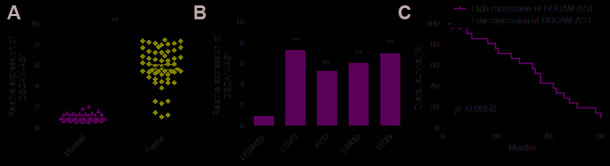
**DSCAM-AS1 was upregulated and correlated with poor prognosis in patients with CRC.** (**A**) qRT-PCR shows the lncRNA DSCAM-AS1 expression level in 56 pairs CRC tissues and non-tumor tissues. (**B**) qRT-PCR shows the lncRNA DSCAM-AS1 expression level in a normal human colon epithelial cell line NCM460 and four human CRC cell lines (LOVO, PKO, SW480 and HT29).(**C**) 56 patients with CRC were divided into two groups based on a median DSCAM-AS1 value and the association of DSCAM-AS1 expression with overall survival was analyzed with a Kaplan–Meier plot. *P* < 0.05, **P < 0.01.

The expression of DSCAM-AS1 in 56 CRC tissues and adjacent normal tissues was detected, and we found a significant increase in its expression in CRC tissues (Figure 1A). We also detected the expression of DSCAM-AS1 in CRC cell lines and found that in 4 CRC-derived cell lines (LOVO, PKO, SW480 and HT29) DSCAM-AS1 expression was significantly higher than in the normal human colon epithelial cell line NCM460 (Figure 1B).

To investigate the correlation between DSCAM-AS1 and clinicopathological features of patients with CRC, the 56 patients were split into two groups based on the median value: DSCAM-AS1 high group and DSCAM-AS1-low group. As shown in Table 1, high DSCAM-AS1 group was associated with advanced clinical stage and lymph node metastasis. In addition, Kaplan-Meier curve and log rank test showed that overall survival (OS) was significantly shorter in the high DSCAM-AS1 expression group relative to low DSCAM-AS1 group (Figure 1C).

**Table 1:** Association of DSCAM-AS1 expression with clinicopathologic factors of 56 patients with CRC.

**Variables**	**No. of cases**	**DSCAM-AS1 expression**		***P* value**
		**High**	**Low**	
Age(years)				*P*=0.4139
<50	22	10	12	
≥50	34	20	14	
Gender				*P*=0.2836
Male	29	18	11	
Female	27	12	15	
TNM stage				***P*=0.0126**
I-II	43	19	24	
III-IV	13	11	2	
Location				*P*=0.1766
Colon	24	10	14	
Rectal	32	20	12	
Lymph node metastasis				***P*=0.0003**
No	41	16	25	
Yes	15	14	1	

Statistical significant results (in bold)

### DSCAM-AS1 knockdown inhibited proliferation of CRC cells

**Figure 2:**
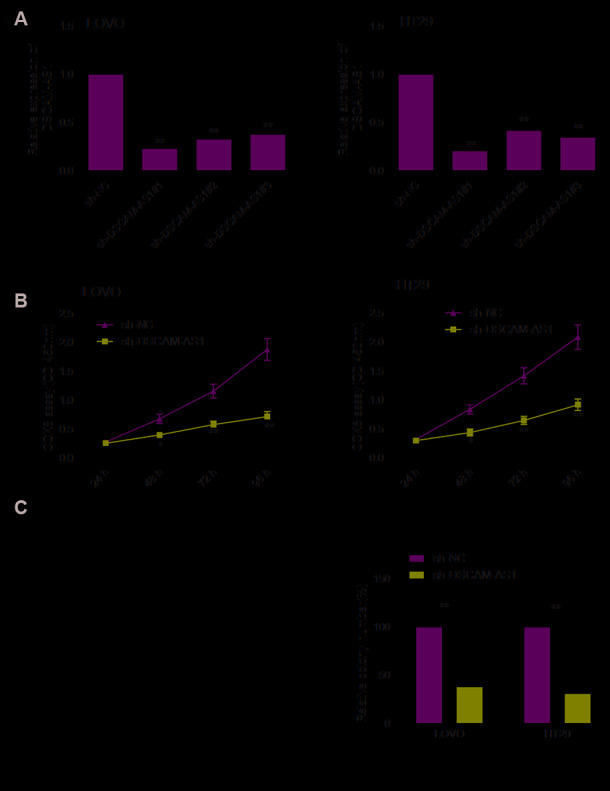
**DSCAM-AS1 knockdown inhibits the proliferation of CRC cells.** (**A**) The knockdown efficiencies of three shRNA against DSCAM-AS1 (sh-DSCAM-AS1#1, sh-DSCAM-AS1#2 and sh-DSCAM-AS1#3) in LOVO and HT29 cells were detected by qRT-PCR analysis. (**B** and **C**) Cell proliferation and colony formation were determined in LOVO and HT29 cells transfected with sh-NC or sh-DSCAM-AS1. *P*< 0.05, **P < 0.01.

To study the role of DSCAM-AS1 in CRC, we performed loss-of-function experiment by downregulating the expression of DSCAM-AS1 in LOVO and HT29 cells using sh-DSCAM-AS1#1, sh-DSCAM-AS1#2 and sh-DSCAM-AS1#3. As seen in Figure 2A, the three shRNAs significantly inhibited DSCAM-AS1 expression in LOVO and HT29 cells. Due to the efficiency of knockdown, we chose sh-DSCAM-AS1#1 as the DSCAM-AS1 down-regulation for subsequent studies, and named as: sh-DSCAM-AS1. CCK8 assay indicated that DSCAM-AS1 knockdown resulted in a significant inhibition of proliferation of LOVO and HT29 cells (Figure 2B). Consistent with this result, depletion of DSCAM-AS1 obviously decreased colony formation of LOVO and HT29 cells (Figure 2C).

### DSCAM-AS1 knockdown inhibited migration and invasion of CRC cells

**Figure 3:**
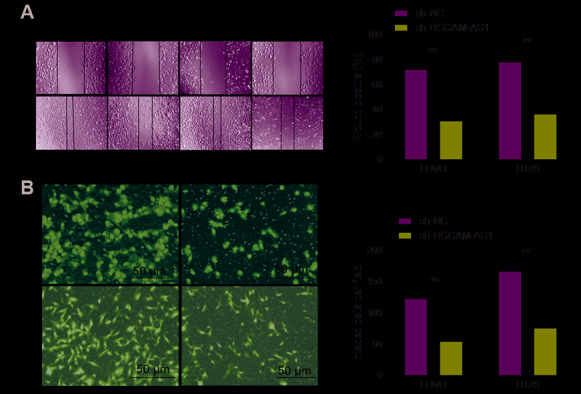
**Knockdown of DSCAM-AS1 inhibits migration and invasion of CRC cells.** (**A**) Cell migration was examined in LOVO and HT29 cells transfected with sh-NC or sh-DSCAM-AS1 by wound healing assay. (**B**) Cell invasion was examined in LOVO and HT29 cells transfected with sh-NC or sh-DSCAM-AS1 by transwell invasion assay. *P* < 0.05, **P < 0.01.

The effect of DSCAM-AS1 knockdown on cell invasion was studied using transwell assay and its effect on migration was determined by the wound healing assay. As seen in Figure 3A, down-regulation of DSCAM-AS1 markedly decreased cell migration ability of LOVO and HT29 cells. DSCAM-AS1 depletion also inhibited the cell invasion ability when compared to sh-NC group (Figure 3B). These indicated that downregulation of DSCAM-AS1 suppressed CRC cell metastasis.

### DSCAM-AS1 is a sponge for miR-384

**Figure 4:**
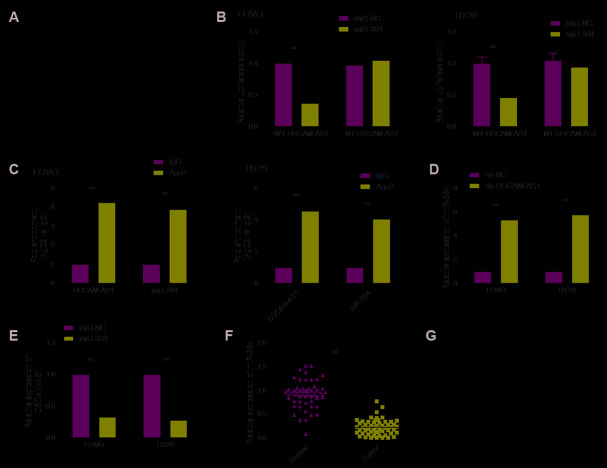
**DSCAM-AS1 acted as a sponge for miR-384.** (**A**) The predicted binding sites of miR-384 on the sequence of DSCAM-AS1(WT-DSCAM-AS1). The target sequences of the DSCAM-AS1 were mutated (MT-DSCAM-AS1). (**B**) Luciferase activity was examined in LOVO and HT29 cells co-transfected with miR-384 mimics or miR-NC, and luciferase reporter vector containing WT-DSCAM-AS1 or MT-DSCAM-AS1. WT: wild-type; MT: mutant-type. (**C**) The interaction between miR-384 and DSCAM-AS1 was determined in LOVO and HT29 cells with RIP assay. (**D**) The expression of miR-384 in LOVO and HT29 cells cells transfected with sh-NC or sh-DSCAM-AS1 was determined by qRT-PCR. (**E**) The expression of DSCAM-AS1 in LOVO and HT29 cells transfected with miR-NC or miR-384 mimic was determined by qRT-PCR. (**F**) qRT-PCR shows the miR-384 expression level in 56 pairs CRC tissues and non-tumor tissues. (**G**) Pearson's correlation analysis between miR-384 expression and DSCAM-AS1 expression in 56 CRC tissues. *P*< 0.05, **P< 0.01.

Emerging studies reveal lncRNA could directly bind to miRNAs and function as a molecular sponge during tumorigenesis [19, 20]. We further investigated the regulatory mechanism of DSCAM-AS1 in CRC. Through bioinformatics analysis using starBase V2 tool, we found that there are miR-384 binding sites in the DSCAM-AS1 sequence (Figure 4A). To test this predication, luciferase reporter assay was carried out and we found that miR-384 over-expression resulted in a significant suppression of the activity of WT-DSCAM-AS1 reporter plasmid in LOVO and HT29 cells (Figure4B). Furthermore, RIP assay demonstrated that DSCAM-AS1 and miR-384 were remarkably clustered in Ago2 immunoprecipitate in comparison with the IgG-pellet, indicating they enriched in the same RNA-induced silencing complex (RISC) (Figure 4C). Furthermore, qRT-PCR analysis showed that DSCAM-AS1 knockdown increased miR-384 expression in LOVO and HT29 cells (Figure4D), while overexpression of miR-384 inhibited DSCAM-AS1 expression in LOVO and HT29 cells (Figure 4E). Moreover, we discovered that there was a downregulation of miR-384 expression in CRC tissues (Figure 4F), and there was negative correlation with DSCAM-AS1 in CRC tissues (Figure 4G).

### DSCAM-AS1 knockdown inhibited the progression of CRC cells by regulating miR-384/AKT3 axis

**Figure 5:**
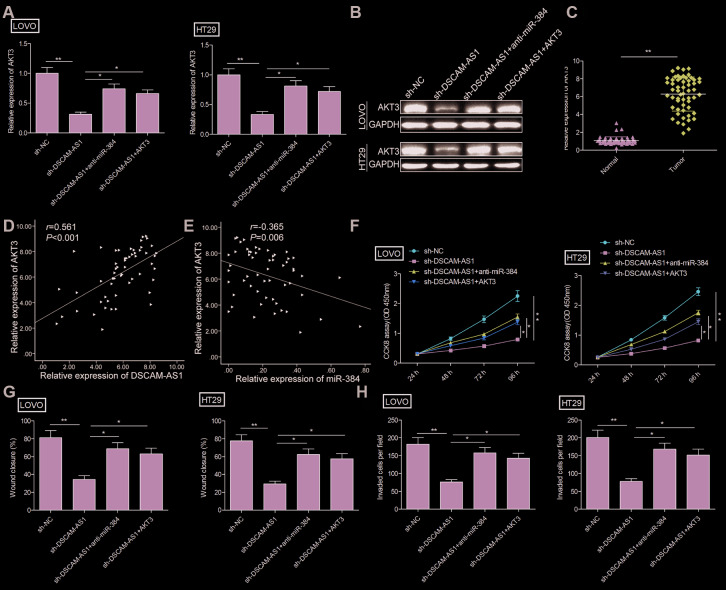
**DSCAM-AS1 knockdown inhibited the progression of CRC cells by regulating miR-384/AKT3 axis.** (**A**, **B**) The expression of AKT3 on mRNA and protein levels was measured in LOVO and HT29 cells after transfection with sh-NC, sh-DSCAM-AS1,sh-DSCAM-AS1+miR-384inhibitor(anti-miR-384)andsh-DSCAM-AS1+overexpression AKT3 plasmid(AKT3). (**C**). RT-PCR shows the AKT3 mRNA expression level in 56 pairs CRC tissues and non-tumor tissues. (**D**) Pearson's correlation analysis between AKT3 expression and DSCAM-AS1 expression in 56 CRC tissue. (**E**) Pearson's correlation analysis between AKT3 expression and miR-384 expression in 56 CRC tissue. (**F**–**H**) Cell proliferation, migration and invasion were determined in LOVO and HT29 cells after transfection with sh-NC, sh-DSCAM-AS1, sh-DSCAM-AS1+anti-miR-384 and sh-DSCAM-AS1+AKT3. *P* < 0.05, **P < 0.01.

Growing evidence suggested that lncRNAs can function as molecular sponges to modulate expression of specific genes by sponging target miRNAs [19, 20]. Studies show that AKT3 is targeted by miR-384 in CRC cells [23]. Here, we investigate whether DSCAM-AS1 could regulate miR-384 expression in CRC cells by sponging miR-384. Our results revealed that DSCAM-AS1 depletion significantly decreased AKT3 expression in LOVO and HT29 (Figure 5A and 5B), while miR-384 inhibitor reversed this trend. In addition, we found that AKT3 expression was increased in CRC tissues (Figure 5C), and its expression was positive correlated with DSCAM-AS1(*r*=0.561; *P*<0.001) (Figure5D), and negative correlated with miR-384(r=-0.365; *P*=0.006) (Figure 5E).

Considering the close correlation between miR-384, AKT3 and DSCAM-AS1, we next evaluated whether the miR-384/AKT3 axis implicates in biological effects by DSCAM-AS1 in CRC cells. To this end, LOVO and HT29 cells were transfected with sh-NC, sh-DSCAM-AS1, sh-DSCAM-AS1+miR-384 inhibitor or sh-DSCAM-AS1+AKT3 overexpression plasmid. Rescue experiments showed that overexpression of AKT3 reversed the effect caused by DSCAM-AS1 knockdown on proliferation, migration and invasion (Figure 5F–5H). Similarly, miR-384 inhibitor partially reversed the inhibitory effect caused by DSCAM-AS1 knockdown in CRC cells (Figure 5F–5H). In summary, these findings suggested that DSCAM-AS1 promoted CRC progression via modulation of AKT3 by acting as a ceRNA of miR-384.

### Knockdown of DSCAM-AS1 impeded CRC tumor growth in nude mice

**Figure 6:**
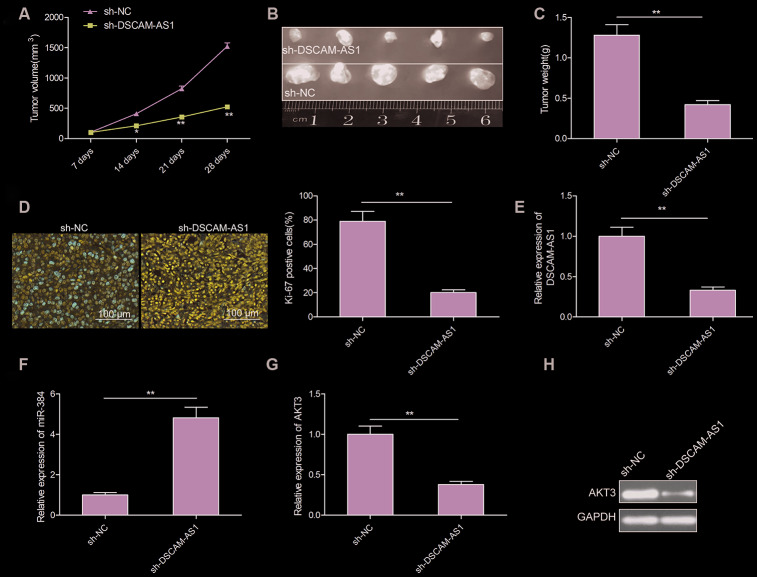
**Knockdown of DSCAM-AS1 suppressed tumor growth *in vivo.*** (**A**) Tumor growth curves were calculated in nude mice after subcutaneously injection of DSCAM-AS1-depletion LOVO cells. (**B**) Representative image of isolated tumor from nude mice. (**C**) The tumor weights were examined in isolated tumor from nude mice. (**D**) The expression of Ki-67 was measured in tumors derived from mice by immunostaining. (**E**, **F**) The expression of DSCAM-AS1 and miR-384 were examined in xenografted tumor by qRT-PCR. (**G**, **H**) The expression of AKT3 on mRNA and protein levels was measured in xenografted tumor. *P*< 0.05, **P < 0.01.

The tumor xenograft assay was done to study the impact of DSCAM-AS1 knockdown on CRC tumor growth *in vivo*. As seen in Figure 6A–6C, nude mice injected with sh-DSCAM-AS1/LOVO cells had smaller tumors, both in volume and weight, when compared to mice injected with sh-NC/LOVO cells. Moreover, we found that the Ki-67 positive cells were significantly decreased in sh-DSCAM-AS1/LOVO group when compared to sh-NC/LOVO group (Figure 6D). We also found that DSCAM-AS1 and AKT3 expression was downregulated, while miR-384 was upregulated in xenograft tumor of sh-DSCAM-AS1/LOVO group when compared to sh-NC/LOVO group (Figure 6E–6H). The *in vivo* data therefore complemented the description about biological role of DSCAM-AS1.

## DISCUSSION

In recent decades, evidence has highlighted that the dysregulation of lncRNAs on CRC could be a leading cause for tumor process [8, 9]. Studies show that DSCAM-AS1 has a role in progression of several cancers, and that it can function as an oncogenic lncRNA in these cancers [10–16]. Although recently studies demonstrated that DSCAM-AS1 expression was upregulated and played a crucial role in CRC, the functional roles and underlying mechanism of DSCAM-AS1 in CRC cells remains largely unknown [17, 18]. We discovered that there is an upregulation of DSCAM-AS1 in CRC tissues and cell lines. Increased DSCAM-AS1 had a positive correlation with advanced clinical stage, lymph node metastasis and poor overall survival, indicating that it may act as an oncogene. Results of loss-of-function experiments revealed that the knockdown of DSCAM-AS1 in CRC cells can inhibit migration, cell proliferation, and invasion *in vitro*, as well as cause suppression of tumor growth *in vivo*, which suggests that DSCAM-AS1 function is required for CRC progression.

It is well known that lncRNAs function as ceRNAs and interact with miRNAs [19, 20]. DSCAM-AS1 was reported to serve as a ceRNA for sponging several miRNAs to regulate the development of tumors [10, 13, 15, 17]. For example, it was reported that DSCAM-AS1 promotes proliferation and decreases apoptosis of breast cancers cells by regulating miR-204-5p/RRM2 axis [15]. Huang et al. revealed the association between DSCAM-AS1 and poor clinical prognosis, and that it contributes to promoting melanoma progression by sponging miR-136 [13]. Ma et al. indicated that DSCAM-AS1 served as a ceRNA of miR-137 and caused regulation of EPS8, bringing about promotion of cell reproduction and suppression of cell apoptosis in tamoxifen resistance breast cancer cells [24]. Through bioinformatics analysis, miR-384 was found to be a potential target of DSCAM-AS1. Previous studies reported that miR-384 functioned as a tumor suppressor in multiple human malignancies [25–27]_._ In CRC, miR-384 expression was significantly downregulated, and overexpression of miR-384 suppressed the CRC growth and metastasis [23, 28]. Our study exhibited the regulatory relationship between DSCAM-AS1 and miR-384 through luciferase reporter activity and RIP assays. Our study also indicated that miR-384 expression was decreased in CRC tissues, which was consistent with previous results [23, 28]. In addition, there was a negative correlation between miR-384 expression and DSCAM-AS1 in CRC. It is important to note that miR-384 inhibitor partially reversed the inhibitory effect caused by DSCAM-AS1 knockdown in CRC cells. Therefore we suggest that DSCAM-AS1 promoted CRC progression by sponging for miR-384. Previous studies showed that DSCAM-AS1 could sponge miR-216b and miR-144-5p in CRC [17, 18]. Linked with our results, DSCAM-AS1 could sponge multiple miRNAs in CRC progression.

Various examples in literature have demonstrated that lncRNA functioned as a ceRNA of miRNA that brings about modulation of depression of miRNA's target gene expression [29]. Previous study showed that AKT3 is targeted by miR-384 in CRC cells [23]. Here, we showed that in CRC cells, down-regulation of DSCAM-AS1 significantly reduced AKT3 expression, while overexpression of AKT3 or downregulation of miR-384 could reserve this trend. Furthermore, AKT3 expression was upregulated, and its expression was positive correlated with DSCAM-AS1 and negative correlated with miR-384 in CRC tissues. Overexpression of AKT3 in CRC cells reversed the effect caused by knockdown of DSCAM-AS1 on proliferation and invasion. These results implied that DSCAM-AS1 acts as a ceRNA of miR-384, bringing about modulation of AKT3 expression, thereby promoting the progression of CRC.

In summary, we identified that lncRNA DSCAM-AS1 is linked to tumor metastasis and poor OS of CRC patients. DSCAM-AS1 promoted the progression of CRC by playing the role of a ceRNA of miR-384 to modulate AKT3 expression. Thus, DSCAM-AS1 has the potential to be a diagnosis marker as well as a therapeutic target for CRC.

## MATERIALS AND METHODS

### Clinical tissues

Harvesting of 56 clinical CRC samples and adjoining normal tissues were carried out at Department of Gastrointestinal Colorectal and Anal Surgery, China-Japan Union Hospital of Jilin University between the time period January 2011 to January 2012. None of the patients had received any anti-tumor therapy before surgery. Written informed consent was acquired from the patients. Research approval was got from “Ethics Committee of Jilin University (Changchun, China).

### Cell culture and transfection

Normal human colon epithelial cell line NCM460 and four human CRC cell lines (LOVO, PKO, SW480 and HT29) were bought from American Type Culture Collection (ATCC; Manassas, VA, USA). Along with supplementation of 10% fetal bovine serum (FBS, Gibco; MA, USA), cells were grown in Dulbecco's modified Eagle's medium (DMEM; Gibco) containing with 100 U/ml penicillin (Invitrogen, CA, USA) and 1 μg/ml streptomycin (Invitrogen). All cells were maintained in a humidified incubator at 37 °C with 5% CO2.

Mimics and inhibitors of miR-384, as well as corresponding negative controls were bought from Gene Pharma (Shanghai, China). CRC cells were transiently transfected with 100 nM miR-384 mimics or inhibitors by lipofectamine 3000 (Invitrogen, USA). Synthesis of three short hairpin (sh)RNA that targeted DSCAM-AS1 (sh-DSCAM-AS1#1, sh-DSCAM-AS1#2 and sh-DSCAM-AS1#3) and the scramble negative control (sh-NC) was done by GenePharma (Shanghai, China), followed by cloning in pGreenPuro™ Vector (System Biosciences, CA, USA) 100 ng shRNA plasmids were transfected into CRC cells by lipofectamine 3000 (Invitrogen, USA) as per company protocol. The selection of stable transfectants was done with G418 (500 mg/ml, Invitrogen). Sequences of shRNAs were presented as listed Table 2.

**Table 2:** Sequences of shRNAs and primers.

**Name**	**Sequence(5’-3’)**
Sequence of shRNAs	
sh-DSCAM-AS1#1	GGAGATCACAGCCAAGGAA
Sh-DSCAM-AS1-#2	CAAAACCACAACAACAACA
sh-DSCAM-AS1#3	GTTAACATTTGGTGTAATTTG
sh-NC	TTCTCCGAACGTGTCACGTTT
Primers used for qRT-PCR	
DSCAM-AS1 Forward	CCAGGAACCAATCCTTACTC
DSCAM-AS1 Reverse	’CCCTAGGGATGTGACCGAAGGA
AKT3 Forward	ATACACGCAAATACACTCC
AKT3 Reverse	’CCCTAGGGATGTGACCGAAGGA
GAPDH Forward	GGGCTGCTTTTAACTCTGGTAAAG
GAPDH Reverse	CCATGGGTGGAATCATATTGG
miR-384 Forward	TGTTAAATCAGGAATTTTAA
miR-384 Reverse	TGTTACAGGCATTATGAA
U6 Forward	CTCGCTTCGGCAGCACA
U6 Reverse	AACGCTTCACGAATTTGCGT

AKT3 overexpression plasmid (pCDNA-3.1) was granted from Dr Li (Jilin University) was transfected into CRC cells using lipofectamine 3000 (Invitrogen) as per company protocol. Transfection efficiency was examined using real time quantitative PCR (qRT-PCR) at 48 h after transfection.

### RNA extraction, reverse transcription and quantitative PCR

Total RNA was extracted with TRIzol reagent (Invitrogen) from CRC tissues and cell lines. The quality and concentration of RNA were assessed at 260/280 nm by the use of a Nanodrop Spectrophotometer (ND-2000, Thermo, USA). With a Prime Script Kit, reverse-transcription of RNA samples(1μg) into cDNA was carried out (Takara, China). Quantitative PCR reactions were done with SYBR Green PCR Kit (Roche, Germany) under the ABI Prism 7500 system (Applied Biosystems, USA). GAPDH was used for normalization of DSCAM-AS1 and AKT3 mRNA, while U6 was used for normalization of miR-384. All primers sequences are shown in Table 2 [12, 23]. 2^−ΔΔCt^ method was used to examine gene expression levels [30].

### Cell proliferation assay

CRC cells proliferating ability was estimated with Cell Counting Kit-8 (CCK-8; Dojindo, Japan). 100 μl of transfected cells (5 × 10^3^ cells per well) were seeded into 96-well plates and cultured at 37 °C in a humidified incubator with 5% CO2. After the following incubation times (24 h, 48 h, 72 h and 96 h), addition of 20 μl CCK-8 solution was carried out followed by incubation for 4 hours. With a microplate reader, the optical density at 450 nm was determined (Multiscan MK3; Thermo Fisher Scientific, MA).

### Wound healing assay

Cell migration determination was carried out by this assay according to a previous a study [31]. Briefly, transfected cells (1 × 10^4^ cells per well) were seeded into 6-well culture plates and grew until full confluence. The wound area was made by scratching cell monolayer with a 100 μl Eppendorf tip. After scratching, the wells were gently washed twice with PBS to remove the detached cells and residual serum, and were cultured in free-serum medium for 24 h. Scratch wounds were imaged the same position at 0 h and 24 h using an Olympus microscope (Tokyo, Japan).

### Transwell invasion assay

Following trypsinization, cells were seeded with Matrigel-coated (BD Biosciences, USA) transwell filters in a 24-well plate (50,000 cells/well) in serum-free medium. The lower chambers contained medium with 20% FBS. Following 24h incubation, fixation of invaded CRC cells were carried out with methanol for 30 min followed by staining for a duration of 15 min with 0.1% crystal violet. The number of invasive cells was determined by counting the stained cells at five fields selected by random with an inverted microscope (Olympus, Japan) at 200 × magnification.

### Bioinformatic analysis and luciferase reporter assay

StarBase2.0 was used to predict the potential binding sites of miRNAs on DSCAM-AS1 [32]. Generation of Wild-type (WT) DSCAM-AS1 with potential miR-384 binding sites were inserted into a luciferase reporter vector psi-CHECK-2 (Promega, USA). In addition, a site-directed mutagenesis kit (Tiangen, Beijing, China) was used to generate a mutated form of this vector termed MT-DSCAM-AS1.Co-transfection of CRC cells with luciferase plasmids and miR-384 mimics or miR-NC were carried out followed by culturing for 48 h. Dual-Luciferase Reporter Assay System (Promega) was used to determine the luciferase activities, following the manufacturer’s instructions.

### RNA immunoprecipitation (RIP) assay

RNA-Binding Protein Immunoprecipitation Kit (Millipore, USA) was used as per manufacturer’s instructions. LOVO and HT29 cells were lysed and treated with RIP buffer with magnetic beads that had undergone conjugation with human anti-human argonaute 2 (Ago2) antibody (Millipore, USA). Mouse IgG (Millipore) was used for negative control. Immunoprecipitated RNA was extracted using TRIzol reagent after the protein was digested using Proteinase K buffer. qRT-PCR was carried out for detection of DSCAM-AS1 and miR-384 as above-mentioned.

### Western blot analysis

Total proteins were extracted from tissues or cultured cells by a radio immunoprecipitation assay buffer (Sigma, St. Louis, MO, USA). Samples were separated in 10% sodium dodecyl sulphate-polyacrylamide gel electrophoresis (SDS-PAGE), and transferred onto a a 0.22μm polyvinylidene difluoride (PVDF) membrane (Merck Millipore, Billerica, MA, USA). Then membrane was incubated with primary antibodies of AKT3 (1:500 dilution; ab32505, Abcam, Cambridge, MA, UK) and GAPDH (1:5000 dilution, ab8245, Abcam), followed by incubation with secondary antibody (1:8000 dilution; Abcam, ab6721). The protein expression was observed using the ECL (electrochemiluminescence) kit (Millipore, Billerica, MA, USA).

### Established of xenograft model

All animal experimental protocols and surgical procedures were approved by the “Animal Care and Use Committee of Jilin University (Changchun, China)”. Ten male athymic nude mice (5-week-old, 18-20g) were obtained from the “Laboratory Animal Center of Jilin University (Changchun, China)”. A total of 2 × 10^6^ LOVO cells that had been stably transfected using either sh-DSCAM-AS1 or sh-NC were implanted subcutaneously into 5-week old nude BALB/c mice (n = 5/group). Using the formula below, tumor volumes were determined every 5 days: Tumor volume = (Length × Width^2^)/2. The mice were sacrificed after a duration of 30 days and the tumors were removed and weighed for further studies.

### Immunohistochemistry (IHC)

The tumors tissues were fixed with 10% neutral buffered formalin and embedded in paraffin. Then Paraffin-embedded tissue sections (4-μm) were deparaffinized, rehydrated and immunostained for detection of Ki-67 (a proliferation marker) expression levels. Staining of slides with Ki-67 antibody (1:400 dilution; ab16667, Abcam) was carried out. EnVision FLEX High pH 9.0 Visualization System (DAKO) was then used, followed by incubating with streptavidin horseradish peroxidase (LSAB kit; Dako, Denmark) and staining with 3, 3-diaminobenzidine (DAB). Staining of sections with hematoxylin was then done after which it was dehydrated, mounted, and photographed with a light microscope (Olympus).

### Statistics analysis

SPSS 19.0 (Armonk, USA) and GraphPad Prism software 5.01 (La Jolla, USA) was used for statistical analysis. To analyze the differences, Student’s t-test and one-way ANOVA was carried out. A *P* value less than 0.05 were considered as significance. Correlations between DSCAM-AS1, AKT3 and miR-384 were conducted by Pearson’s correlation. Kaplan-Meier curve and log rank test was utilized to determine survival rate. All data was shown as mean ± standard deviation (SD) with at least 3 replicates measurements.
